# Expression Profiling of Glioblastoma Cell Lines Reveals Novel Extracellular Matrix-Receptor Genes Correlated With the Responsiveness of Glioma Patients to Ionizing Radiation

**DOI:** 10.3389/fonc.2021.668090

**Published:** 2021-05-25

**Authors:** Rodolfo Bortolozo Serafim, Patrick da Silva, Cibele Cardoso, Luis Fernando Macedo Di Cristofaro, Renato Petitto Netto, Rodrigo de Almeida, Geovana Navegante, Camila Baldin Storti, Juliana Ferreira de Sousa, Felipe Canto de Souza, Rodrigo Panepucci, Cristiano Gallina Moreira, Larissa Siqueira Penna, Wilson Araujo Silva, Valeria Valente

**Affiliations:** ^1^ School of Pharmaceutical Sciences, São Paulo State University (UNESP), Araraquara, Brazil; ^2^ Center for Cell-Based Therapy (CTC), Regional Blood Center of Ribeirão Preto, Ribeirão Preto, Brazil; ^3^ Department of Genetics, Ribeirão Preto Medical School, University of São Paulo (USP), Ribeirão Preto, Brazil; ^4^ Radiation Oncology Branch, National Cancer Institute, National Institutes of Health (NIH), Bethesda, MD, United States

**Keywords:** glioblastoma, GBM cell lines, expression profiling, extracellular matrix, ECM-receptors, radioresistance

## Abstract

Glioblastoma (GBM) is the most lethal and frequent type of brain tumor, leading patients to death in approximately 14 months after diagnosis. GBM treatment consists in surgical removal followed by radio and chemotherapy. However, tumors commonly relapse and the treatment promotes only a slight increase in patient survival. Thus, uncovering the cellular mechanisms involved in GBM resistance is of utmost interest, and the use of cell lines has been shown to be an extremely important tool. In this work, the exploration of RNAseq data from different GBM cell lines revealed different expression signatures, distinctly correlated with the behavior of GBM cell lines regarding proliferation indexes and radio-resistance. U87MG and U138MG cells, which presented expressively reduced proliferation and increased radio-resistance, showed a particular expression signature encompassing enrichment in many extracellular matrix (ECM) and receptor genes. Contrasting, U251MG and T98G cells, that presented higher proliferation and sensibility to radiation, exhibited distinct signatures revealing consistent enrichments for DNA repair processes and although several genes from the ECM-receptor pathway showed up-regulation, enrichments for this pathway were not detected. The ECM-receptor is a master regulatory pathway that is known to impact several cellular processes including: survival, proliferation, migration, invasion, and DNA damage signaling and repair, corroborating the associations we found. Furthermore, searches to The Cancer Genome Atlas (TCGA) repository revealed prognostic correlations with glioma patients for the majority of genes highlighted in the signatures and led to the identification of 31 ECM-receptor genes individually correlated with radiation responsiveness. Interestingly, we observed an association between the number of upregulated genes and survivability greater than 5 years after diagnosis, where almost all the patients that presented 21 or more upregulated genes were deceased before 5 years. Altogether our findings suggest the clinical relevance of ECM-receptor genes signature found here for radiotherapy decision and as biomarkers of glioma prognosis.

## Introduction

Glioblastoma (GBM) is the most common and aggressive primary brain tumor in adults. The current protocols for treating GBM involve surgical resection followed by chemotherapy and radiotherapy, but the rate of patient survival is only 14 months (1). The poor prognosis accrues mainly from the resistance of GBM cells against chemical and radiotherapeutic agents, along with the abrupt increase in proliferation acquired during tumor progression. The extensive interaction of tumor cells with the extracellular matrix (ECM) favors invasiveness and brain infiltration, preventing the cure even after extensive surgical resection (2). In face of this scenario, standard treatment does not restrain recurrences and about 80% of relapses are located at the resection margin, which is the site predominantly affected by higher doses of radiation (3). Also, GBM resistance is notably correlated with the high percentage of cancer stem cells (CSC) population usually found in these tumors (4). CSC responds to genotoxic agents in an adaptive manner and usually survives inside the therapeutic environment, quickly regenerating the tumor after treatment cessation and supporting relapses in a few months (5). Thus, a better understanding of tumor cells’ response to irradiation is crucial for the comprehension of GBM aggressiveness.

The accelerated proliferation of GBM cells also drives the pronounced complexity of these tumors that typically encompasses a huge genetic diversity along with a heterogeneous microenvironment. The presence of hypoxic and hyper-perfused regions, redox gradients and different pro-inflammatory cytokines sustain a dynamic environment, promoting both the proliferative and infiltrative phenotypes (6). Also, a diversity of ECM proteins interacts with tumor and stromal cells and plays a central role in cellular communication, supplying growth factor production, migration and invasion (7, 8). The ECM is usually remodeled in tumor tissues and promotes desmoplasia, which is characterized by an increase in total levels of fibrillar collagen, fibronectin, proteoglycans, and tenascin C. These changes have been associated with tumor *stiffening*, which is a more rigid and resistant state that acquires at least 1.5-fold higher resistance to mechanical stress than the surrounding normal tissue. The *stiffening* state can promote epithelial to mesenchymal transition (EMT) that is also correlated with drug resistance and it is hypothesized to contribute to transformation of cancer cells in CSC (9).

Irradiation, the major therapeutic approach used to handle GBM, also produces important changes in the tumor microenvironment that may support resistance, proliferation and recurrence, such as: increased oxidative stress, hypoxia, neuroinflammation, altered expression of adhesion molecules, senescence induction and neo-angiogenesis. The high levels of radiation-induced reactive oxygen species (ROS) promote the remodeling of collagen and proteoglycans, activation of cellular proteases and changes in the cytoskeleton, contributing to development of senescent phenotypes (6). Metabolic changes also occur in tumor microenvironments in response to radiation-induced oxidative stress, such as increased production of antioxidant peptides and hypoxia generation. Radiation-induced stabilization/activation of HIF-1 (Hypoxia Inducible Factor 1) triggers protective processes by regulating downstream target genes that can stimulate immunosuppressive and anti-apoptotic responses (10). Another important aspect is the radiation-induced bystander effect, such as mitochondrial dysfunction, production of persistent or irreparable DNA damage, DDR (DNA damage response) activation, irreversible cell cycle arrest, and also the cellular senescence (6). Furthermore, GBM cells were reported to be able to constitutively activate senescence by blocking cyclin-dependent kinase inhibitors, for example p16 and p21 (11), which favors the release of pro-inflammatory signaling molecules and proteolytic enzymes (12).

Thus, the exposure of GBM to radiation can affect the composition of the ECM, altering the tumor proliferation and infiltration capabilities. These phenotypes are supported by the overexpression of diverse ECM proteins, including structural components (brevican, vitronectin, tenascin C, hyaluronin, lysyl oxidase), degradation enzymes (Matrix metalloproteinases) (13) and glioma-matrix interactors (ICAM-1, DDR-1, Integrins) (14, 15). Altogether, these data disclose that the relationship between tumor cells and the ECM supports the particularly infiltrative phenotype of GBM that is strongly correlated with radioresistance (16). As radiotherapy remains a primary treatment modality for gliomas, it is of great interest to better understand the acquisition of radioresistance of glioma cells and the targets to modify their tolerance to radiation. Different GBM cell lineages, such as U87MG, T98G, U251MG, are frequently used in studies of radioresistance (17–20). From clonogenic survival assays, the T98G cell line showed higher radioresistance when compared to the U87MG lineage, consistent with the lower levels of ATM kinase in U87MG cells (18). Differently, another study showed that U87MG presents higher resistance against irradiation (IR) than T98G cells and, in both cell lines, the radioresistance profile was correlated with mTOR/AKT activity, which is an important pathway not only for cell survival but also for the maintenance of astrocytic characteristics (20). It was also demonstrated that U87MG cells are more radioresistant than the U251MG lineage. This behavior was associated with the cell cycle dynamics, which was prevalent in G1/S phases for U87MG and in G2/M for U251MG cells, and with the expression of the APE1 (Human Apurinic Endonuclease 1), an enzyme involved in base excision repair (19). Thus, although several studies have investigated radioresistance of GBM cell lines, the literature is divergent and a comprehensive analysis of gene expression profiling associated with cells’ response to IR is still missing. These data would be of great importance since they will permit a wider understanding of the genetic heterogeneity of these cell lines and the molecular basis of the resistant phenotype of GBM cells.

In this work we have explored gene expression profiles of different GBM cell lines that present distinct phenotypes regarding radiation sensibility, aiming the identification of genetic characteristics correlated with radiation responsiveness. Using RNAseq data from five different GBM cell lines, we identified distinct profiles of deregulated genes in radioresistant *versus* sensitive cells. The ECM-cell interaction pathway was predominantly altered in irradiation-resistant cells (U87MG and U138MG), while the DNA damage response/DNA repair pathways were preferentially altered in irradiation-sensitive cells (T98G and U251MG). Consistently, T98G and U251MG cell lines presented the highest proliferation ratios, which is associated with replicative stress and is known to promote constitutive activation of DNA damage signaling. Among the genes positive or negatively associated with radioresistance, integrins were noteworthy. Furthermore, two integrins remarkably overexpressed in IR-resistant cells, integrin-α5 (ITGA5) and integrin-β1 (ITGB1), were validated by western blot and were also induced after IR treatment, confirming their enrollment in IR responsiveness. Importantly, we have also found 31 genes of the ECM-receptor interaction pathway, out of 83, correlated with poor responsiveness of patients to radiation treatment. Altogether, these data provide additional support to the enrollment of ECM-deregulation in the acquisition of IR-resistance and endorse the suitability of these cell lines for studies aiming the characterization of the genetic basis of radioresistant and/or proliferative phenotypes of GBM cells.

## Methods

### Cell lines, Culture Conditions, and Treatment With Ionizing Radiation

The U87MG, U138MG, U251MG, and T98G cell lines were obtained from the American Type Culture Collection in 2010. U343MG cells were obtained from the laboratory of Prof. Carlos Gilberto Carlotti Junior, FMRP-USP, also in 2010. The ACBRI-371 non-tumor astrocytes were obtained from Cell Systems and gently provided by professor Elza Tiemi Sakamoto Hojo (FFCLRP-USP). Cells were grown in Dulbecco’s Modified Eagle Medium (DMEM, Thermo Fisher Scientific) supplemented with 10% fetal bovine serum (Thermo Fisher Scientific) and incubated at 37°C and 5% CO_2_. The cells were analyzed monthly by immunofluorescence after DNA staining for Mycoplasma detection. Cell line authentication was performed using STR profiling with the GenePrint^®^ 10 System (Promega) following fabricant’s instructions. Authentication was done and validated the identity of U87MG and T98G cell lines in 2017, and the majority of experiments were conducted in the following 6 months. Cells were treated with ionizing radiation at a dose exposure rate of 10 Gy (X-ray irradiator RS-2000, Rad Source) three times every 48 hours for cytotoxicity assay and at a single dose of 10 Gy for apoptosis/cell death and western blot analysis. To choose the radiation regimen, we carried out several previous experiments (data not shown) using the MTT essay to identify a radiation dose that was able to increase cell death ratios and was not excessively toxic for the cell lines studied here.

### Cytotoxicity Assay, Cell Death Analysis, and Growth Curve

The cytotoxicity assay was carried out using the MTT (3- (4,5-dimethylthiazol-2-yl) -2,5-diphenyltetrazolium bromide) method. 500 cells were seeded in 96-well plates, grown for 24 hours and then irradiated three times every 48 hours. The MTT assay was performed 48 hours after the third irradiation treatment. The medium was removed and exchanged for 100 µL of fresh culture medium to cytotoxicity evaluation. Then, 10 µl of 12 mM MTT (3- (4,5-dimethylthiazol-2-yl) -2,5-diphenyltetrazolium bromide) was added over 3 hours at 37°C and 5% CO_2_. After incubation, the crystals were diluted with isopropanol and read on plate reader at 570 nm. The data were analyzed by One-way ANOVA test. Differences were considered significant when p < 0.05. Apoptosis and effective cell death were measured using annexin V and propidium iodide staining. 8x10^4^ cells were seeded in 12 well plates, grown for 24 hours and irradiated. 48 hours after IR treatment, the cells were harvested, incubated with annexin V for 15 minutes and propidium iodide was added to a final concentration of 2 ng/µL. The cell suspension was immediately analyzed in a flow cytometer (FACSCanto, BD Biosciences). The data showed the average of three independent experiments. The results were analyzed by Kruskal–Wallis test (analysis of variance, ANOVA). Differences were considered significant when p < 0.05. For the growth curve, 1x10^3^ cells were seeded in 96 well plates and DNA content evaluated every 24 hours for 6 days. At each analysis point, the culture medium from each well was withdrawn, washed with PBS 1x, and fixed with 70% ethanol for 10 minutes. After, the DNA of the cells was stained with 0.05% crystal violet solution for 15 minutes. Next, the cells were washed and 10% acetic acid solution was added for 30 minutes to read on plate reader at 570 nm. The data were analyzed by Two-way ANOVA test. Differences were considered significant when p < 0.05. The statistical significance was represented by *p<0.05, **p<0.001 and ***p<0.0001. Graphs were plotted with GraphPad Prism 4.0 software.

### RNA Extraction and Quantitative RT-PCR

RNA extraction was performed with RNeasy RNA extraction Kit (Qiagen) following the manufacturer’s instructions. RNA concentration and purity were determined in the spectrophotometer at 260–280 nm. The RNA was treated with DNAse I (Invitrogen) in the presence of an RNAse inhibitor (RNAseOUT; Invitrogen). Then, cDNA synthesis was performed using the High Capacity Kit (Applied Biosystems), also following the manufacturer’s instructions. qPCR reactions were performed in the 7500 RealTime PCR System (Applied Biosystems) using Power SYBR Green PCR Master Mix (Applied Biosystems), according to manufacturer’s recommendations. As previously described, HPRT was used as the control of constitutive expression. The calculations of relative expression were based on the 2^−ΔΔCT^ equation (21). Statistical analyzes were performed with the One-way ANOVA test, using the GraphPad Prism 4.0 software.

### SDS-Polyacrylamide Gel Electrophoresis and Western Blot

Protein samples were separated by electrophoresis in SDS-polyacrylamide gel (6%) and transferred to nitrocellulose membrane. The membrane was blocked with a TBST buffer (1X TBS, 0.25% Tween-20) containing 5% skimmed milk at room temperature for 60 minutes under stirring. The primary antibodies used were: anti-ITGA5 (Cell Signaling, #98204) at dilution 1:1000, anti-ITGB1 (Cell Signaling, #4706) at dilution 1:1000, anti-tubulin (ABCAM, ab7291) at dilution 1:10000, anti-phospho-AKT (Cell Signaling, #4060) at dilution 1:2000, anti-AKT (Cell Signaling, #4691) at dilution 1:1000 and anti-GAPDH (Cell Signaling, #5174) at dilution 1:1000. Images were captured by ChemiDoc™ Imaging System from Bio-Rad.

### Pathway Enrichment and Patient Survival Curves

In order to identify cellular pathways enriched in treatment-resistant *versus* to sensitive GBM cells, we analyzed RNA sequencing data from different GBM cell lines that was previously generated in our laboratory (PRJNA631805). Genes presenting padj ≤ 0,0001 and log fold change > 2 in U87MG and/or U138MG cells (radio-resistant) compared to T98G and/or U251MG cells (radio-sensitive) were considered as differentially expressed. The list obtained for each comparison (**Supplementary Table 1**) was subjected to pathway analysis by KEGG through the website www.webgestalt.org (**Supplementary Table 2**). Pathway enrichment graphs were plotted with GraphPad Prism 4.0 software. For a refined understanding of expression variations among the genes included in enriched pathways, gene expression fold-changes of each GBM cell line relative to non-tumor astrocytes (ACBRI-371) were calculated and illustrated as heatmaps. Thus, gene expression levels in the different GBM cells were estimated considering ACBRI-371 cells as a reference.

We used the data available in The Cancer Genome Atlas (TCGA) database (https://www.synapse.org/#!Synapse:syn2812961) for survival analysis (**Supplementary Table 3**). To access the differences in survival based on ECM genes expression, we choose the best cutoff in expression value of each gene by calculating receiver operating characteristic (ROC) (22) curves for death incidence by time. Survival curves were plotted by the Kaplan-Meier method and compared by the log-rank test. Differences were considered significant when p < 0.05. Graphs were plotted with GraphPad Prism 4.0 software.

## Results

### IR-Resistant GBM Cell Lines Show Higher Expression of ECM Genes and the Interacting Receptors

**Figure 1:**
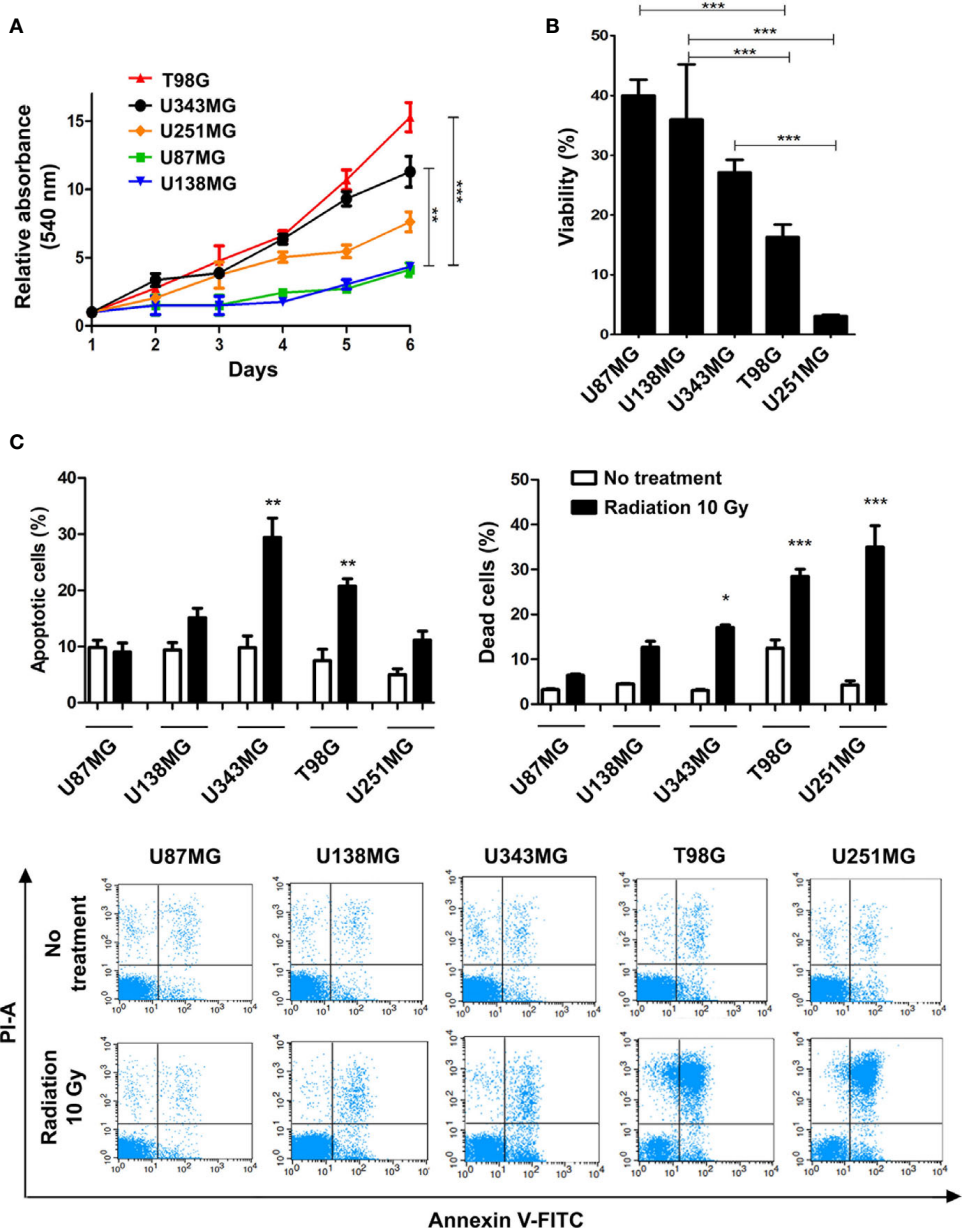
Proliferation and ionizing radiation resistance analysis in GBM cell lines. **(A)** Proliferation assay of T98G, U343MG, U251MG, U87MG, and U138MG. **(B)** Cytotoxicity assay of GBM cells after radiation treatment. **(C)** Quantification of apoptosis and cell death of GBM cells after irradiation are shown in the upper graphics and representative images of flow cytometry experiments are presented in the lower panel. Error bars represent the SEM of 3 independent experiments. Graphs were plotted with GraphPad Prism 4.0 software. *p < 0.05, **p < 0.001, and ***p < 0.0001.

Here we profiled the sensitivity of different GBM cells to ionizing radiation (IR) in order to search for correlations between cell behavior and global gene expression patterns. The panel of cell lines analyzed included U87MG, U138MG, U251MG, U343MG and T98G, which show distinct proliferation indexes. U343MG and T98G cell lines proliferated significantly faster when compared to U87MG or U138MG, showing nearly 3.5 and 5-fold increment in cell number after six days of growing. U251MG exhibited intermediate proliferation rates, significantly differing only from T98G cells that showed 2-fold increment after six days of growing (**Figure 1A**). Cell viability was initially evaluated after three rounds of IR exposure, at a dose of 10 Gy in intervals of 48 hours. Two days after the last treatment, U251MG and T98G cultures showed only 5% and 15% of viable cells respectively, while U87MG and U138MG revealed approximately 40% of viability (**Figure 1B**). These data suggested that U87MG and U138MG cells present higher resistance against IR. However, this experiment does not inform if IR was causing cytotoxicity or a cytostatic disturb in the affected cells. To investigate each of these options were occurring, we evaluated apoptosis and cell death indexes after one round of IR exposure. Again, U87MG and U138MG cells did not exhibit any significant increment in apoptosis or cell death, while T98G and U251MG cultures presented increase only in effective cell death levels, showing 30% and 40% of dead cells, respectively (**Figure 1C**).

**Figure 2:**
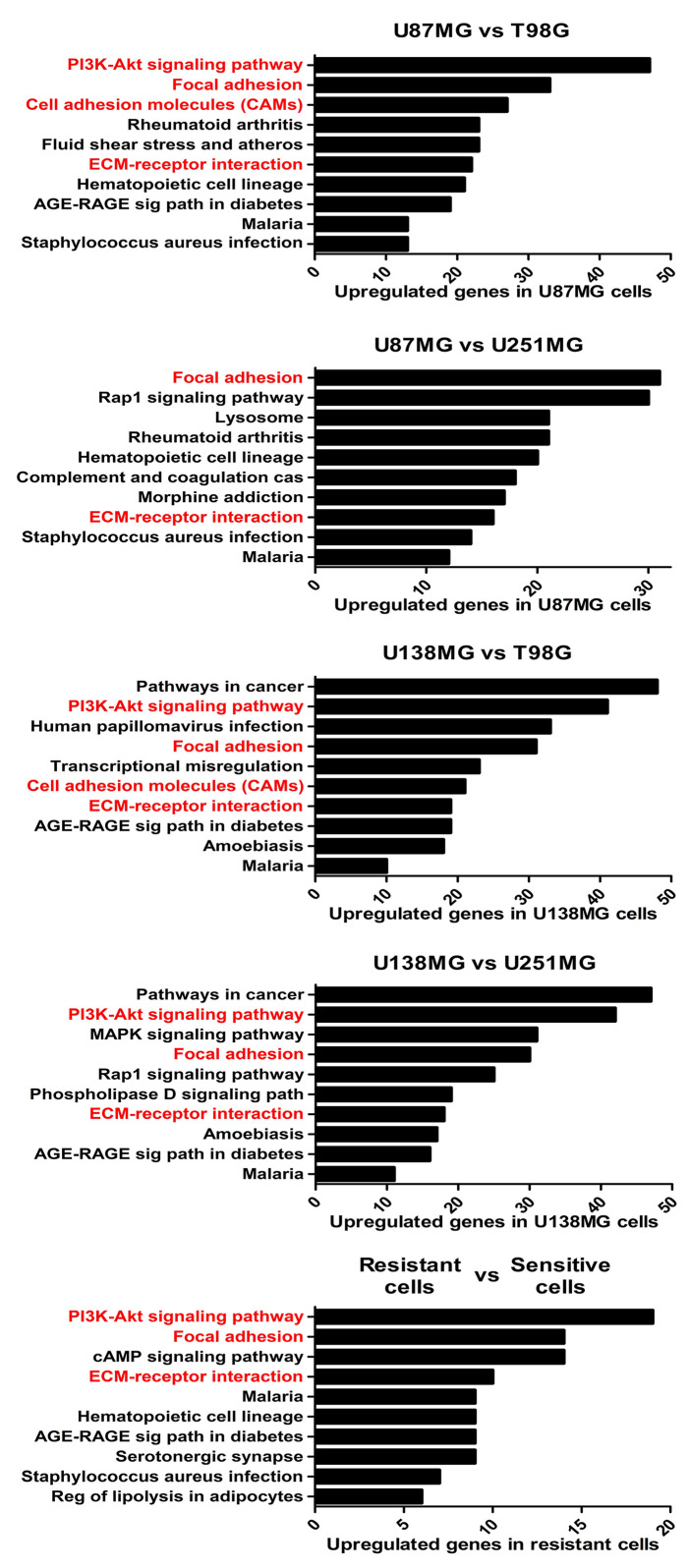
The enriched KEGG pathways in treatment-resistant cells in comparison to sensitive cells. All genes with padj ≤ 0,0001 and log fold change > 2 in U87MG and/or U138MG cells compared to T98G and/or U251MG cells were subjected to pathway analysis by KEGG. The pathways related to cell-extracellular matrix interaction were highlighted in red. A False Discovery Rate (FDR) ≤ 0.05 was used as a threshold to select significant pathways. Graphs were plotted with GraphPad Prism 4.0 software.

**Figure 3:**
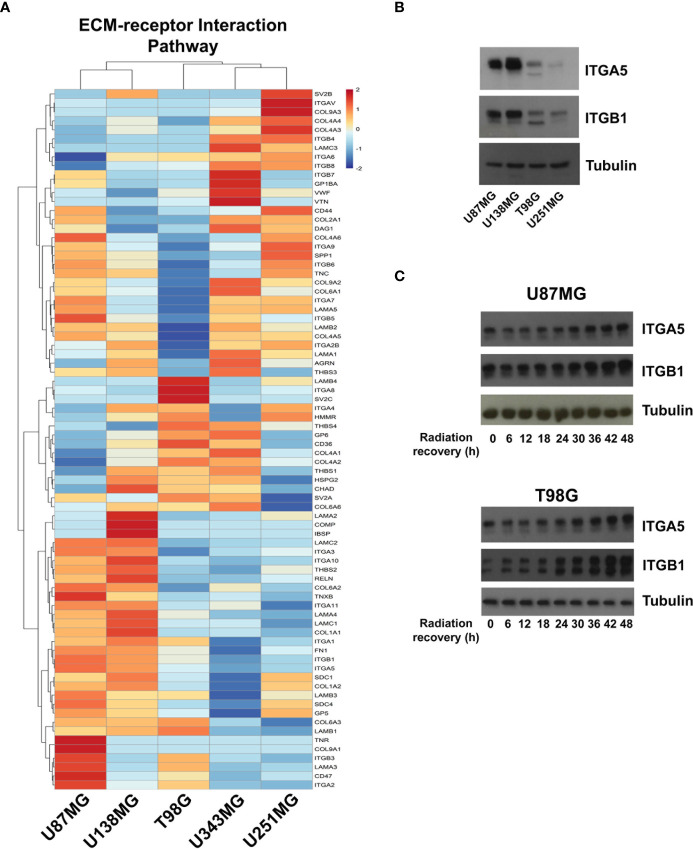
Expression profile of the ECM-receptor interaction genes in GBM cell lines. **(A)** Heatmap of the relative expression of genes enriched in the ECM-receptor interaction pathway, estimated expression in each GBM cell line relative to non-tumor astrocytes (ACBRI-371) are shown. **(B)** Western blot validating ITGA5 and ITGB1 expression in U87MG, U138MG, T98G, and U251MG cell lines. **(C)** Protein samples were collected 0, 6, 12, 18, 24, 30, 36, 42, and 48h after irradiation treatment with 10 Gy and the ITGA5 and ITGB1 protein levels assessed by western blot. The two bands detected for these integrins correspond to the precursor (upper band) and mature (lower band) forms of the protein, and variations are commonly observed depending on the cell line analyzed.

To better understand the molecular mechanisms underlying the competence of these cells in handling genotoxic insults and replicative stress, we further examined global transcriptional profiles available in our laboratory (23). RNAseq analysis revealed a collection of genes differentially expressed in each cell line analyzed when comparing resistant *versus* sensitive cells (**Supplementary Table 1**). Interestingly, IR-resistant cells presented higher expression levels of several ECM components and their related receptors (**Figure 2** and **Supplementary Table 2**). These pathways did not appear enriched when we evaluate the genes increased in radiation sensitive cell lines (**Supplementary Figure 1**). **Figure 3A** shows the relative expression levels of the ECM-receptor interaction genes that significantly varied among GBM cell lines, using non-tumor astrocytes as a reference. Although each cell line revealed a particular set of altered genes, we also observed that the resistant cells (U87MG+U138MG) share several overexpressed genes belonging to the ECM-receptor pathway, indicating promising radio-resistance biomarkers (**Supplementary Table 2**). Additionally, resistant cells usually show overexpression of both, receptors and their ligands simultaneously, while in sensitive cells the overexpression is found only in either receptor or ligand (**Supplementary Table 4** and **Supplementary Figure 2**). These data suggested that the downstream PI3K signaling pathway would not be effectively activated in IR-sensitive cells. Alterations observed in the heatmap of **Figure 3A** were validated by qPCR for a set of five genes arbitrarily selected, namely: ITGA3, ITGA5, ITGA8, LAMB1 and LAMA2. The expression levels of ITGA3 and ITGA5 were significantly higher in U87MG and U138MG cells, ITGA8 and LAMB1 were increased in T98G, and U138MG showed higher levels of LAMA2 (**Supplementary Figure 3**), confirming RNAseq analysis consistency. Two genes presenting increased levels in the resistant cells, ITGA5 and ITGB1, were also examined by western blot (**Figure 3B**) and protein overexpression was confirmed. Since ITGB1 and ITGA5 have been previously described as key players in the acquisition of radioresistance by GBM cells (24–27), we can presume a strength confidence in the correlation between the set of genes identified in our study with IR resistance. Once ITGA5/ITGB1 were also described as responsive to IR, we evaluated their expression levels after IR-treatment. **Figure 3C** shows that both analyzed receptors presented increased protein amounts between 24 and 48 hours after treatment, confirming the requirement of these proteins for radiation responsiveness. Therefore, the endogenous high levels observed in resistant cells and the additional induction promoted by IR, corroborate they indeed play an important role in cellular response to IR, and support that all genes identified here are potentially enrolled in radioresistance acquisition by GBM cells.

Several ECM genes are able to activate the PI3K signaling pathway as a survival mechanism. Thus, we evaluated whether AKT inhibition would sensitize U87MG and U138MG cells to radiation. We observed that AKT levels did not vary between the evaluated cell lines (**Supplementary Figures 4A, B**), however, U87MG and U251MG cells showed higher ratios of AKT phosphorylation at the position S473 (**Supplementary Figure 4B**). Thus, to assess the effects of AKT inhibition in IR resistance, cells were treated with the potent pan-AKT kinase inhibitor GSK690693, for 4 hours followed by irradiation with a dose of 10 Gy. After 48 hours, we observed that the AKT inhibitor was able to increase IR-sensibility of T98G cells at all concentrations evaluated, of U251MG cells at 20 and 30 µM and of U138MG cells only at 30 µM. Although T98G and U251MG showed higher amounts of dead cells when receiving radiation combined with the inhibitor, we did not see any increase in cell death levels for U87MG and a only slight increase for U138MG in the highest concentration of the inhibitor. This result suggested that AKT is not essential for the elevated resistance presented by U87MG and U138MG cell lines. Once other kinases, such as AMPK and DAPK3, might also be affected by the pan-inhibitor we utilized, we could speculate that these kinases, likewise, do not favor sensitization of the resistant GBM cell lines. However, the effect of the combined treatment was shown to be more effective in U251MG cells, indicating that cell lines with reduced amount of upregulated ECM genes are more dependent on the PI3K pathway. Altogether, these data suggest that the set of 31 ECM-receptor genes we found overexpressed in the resistant cell lines activates an extensive pro-survival signaling network that work collectively to sustain IR-resistance in GBM cells.

### Alterations in DNA Repair Pathways Are Enriched in the GBM Cell Lines Presenting Higher Proliferation

**Figure 4:**
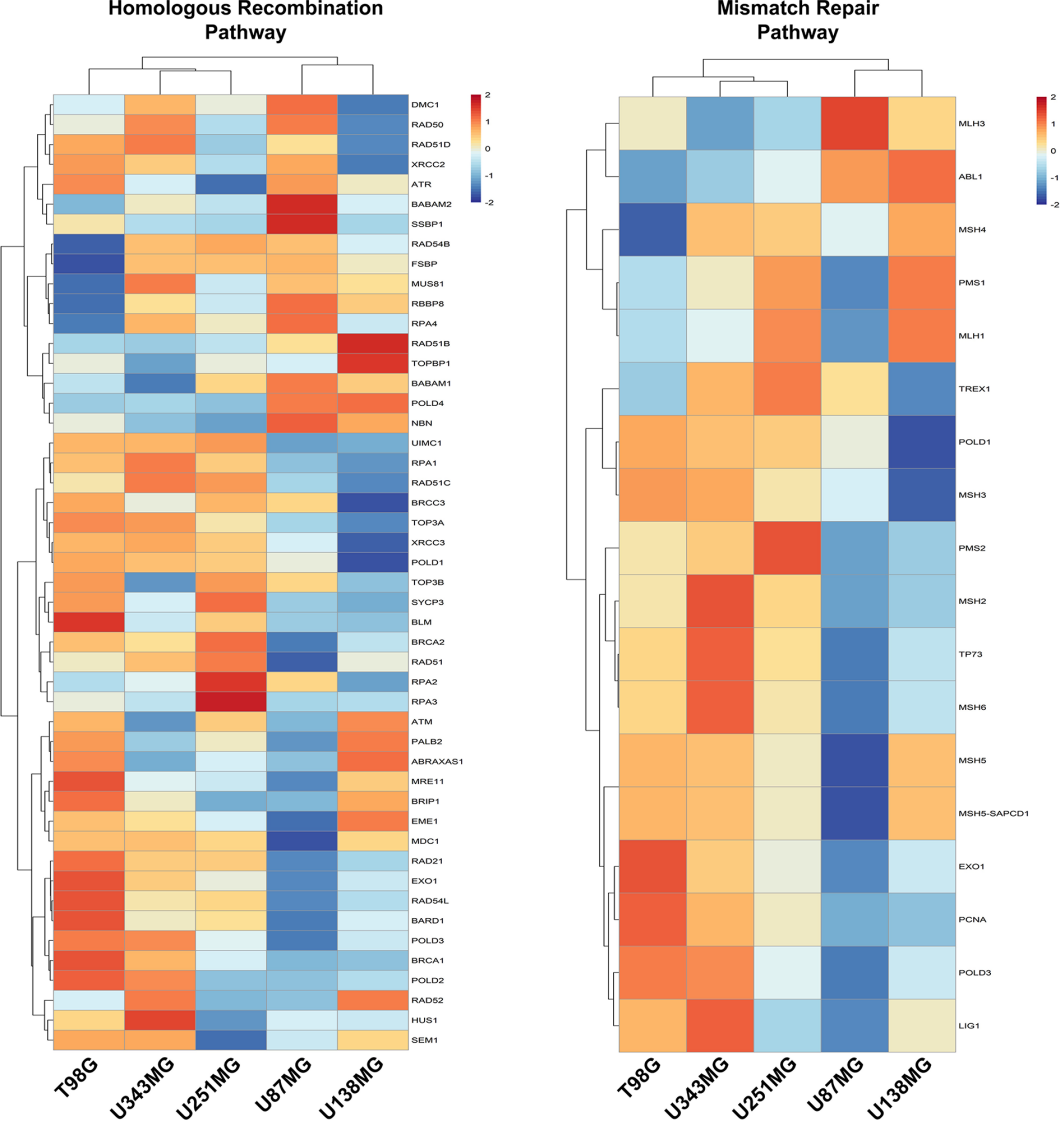
Homologous Recombination and Mismatch Repair pathways are enriched in proliferative GBM cell lines. Heatmaps representative of the expression of genes from the Homologous Recombination and Mismatch Repair pathways were generated with the data from each GBM cell line relative to non-tumor astrocytes (ACBRI-371).

We also investigated if alterations in the expression of DNA repair genes exhibit any association with resistance to ionizing radiation. Actually, we did not observe correlations between variations in the expression of DNA repair genes and IR resistance among the different cell lines analyzed. Also, we observed that these pathways, mainly the Mismatch Repair (MMR) and the Homologous Recombination (HR) pathways, are enriched in cells that showed higher proliferative potential (**Figure 4** and **Supplementary Figure 5**). To confirm the association between DNA repair genes expression and proliferation activity in GBM samples, we searched for correlations between the expression of MKI67, and MMR and HR genes, using the TCGA (The Cancer Genome Atlas) data. We observed that 64.7% and 46.8% of the genes from the MMR and HR pathways show a meaningful Spearman correlation with MKI67 (>0.5), which is comparable with the correlation exhibited by 75.8% of the genes belonging to the DNA replication machinery. Although we did not observe enrichment for genes of the Nucleotide Excision Repair (NER), Base Excision Repair (BER), and Non-Homologous End-Joining (NHEJ) repair pathways in the more proliferative cell lines, these pathways also showed association with MKI67, but at lesser extent, of 37.5%, 29.4% and 21.4%, respectively (**Supplementary Table 5**). These data indicated a tougher association of DNA repair alterations with the proliferative activity than with the radio-resistance.

### ECM-Receptor Signature Derived From IR-Resistant Cells Correlate With Radiation Responsiveness of LGG Patients

**Figure 5:**
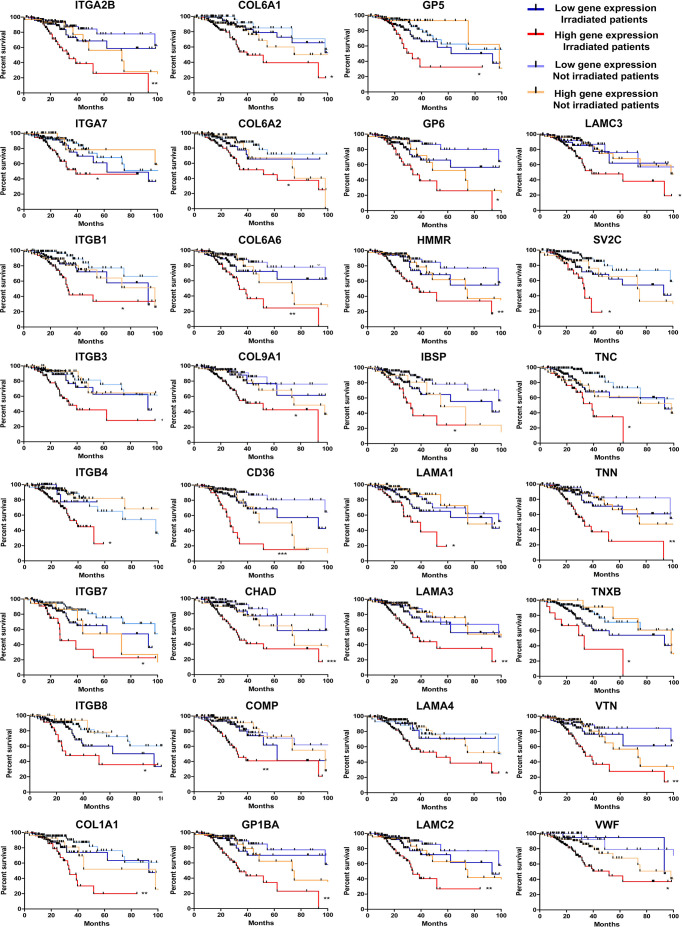
High levels of 31 ECM-receptor interaction transcripts are correlated with poor survival prognosis of radiation-treated patients. Kaplan Meier survival curves for LGG patients according to expression levels of the ECM-receptor interaction genes in the tumors. Patients were divided into four groups: i) untreated patients whose tumors expressed low levels of the analyzed gene; ii) untreated patients whose tumors expressed high levels of the analyzed gene; iii) irradiated patients whose tumors expressed low levels of the analyzed gene; iv) irradiated patients whose tumors expressed high levels of the analyzed gene. *p < 0.05, **p < 0.001, and ***p < 0.0001. The P-values were obtained from a log-rank test. Graphs were plotted with GraphPad Prism 4.0 software.

Next, we seek to understand if the high levels of the ECM-receptor genes were correlated with IR response of lower-grade glioma (LGG) patients from the TCGA database, for which RNAseq data and clinical information were publicly accessible. We have chosen the LGG cohort due to availability of gene expression data and treatment protocols for the majority of samples characterized, whereas for the GBM cohort, treatment regimens are missing for many cases. For this analysis, we separated the patients into 4 groups: i) untreated patients whose tumors expressed low-levels of the analyzed gene; ii) untreated patients whose tumors expressed high-levels of the analyzed gene; iii) irradiated patients whose tumors expressed low-levels of the analyzed gene; iv) irradiated patients whose tumors expressed high-levels of the analyzed gene. Initially, the ROC (Receiver Operating Characteristic) curve was constructed to define the threshold between low and high levels of expression for each target gene (**Supplementary Table 6**). Then, the survival of patients with low or high expression was evaluated. We observed that the increased expression of 31 ECM-receptor genes was correlated with worse prognosis in irradiated patients (**Figure 5**). Importantly, patients selected for radiation treatment show an intrinsic poorer survival prognosis than patients not selected for treatment (**Supplementary Figure 6**), but inside the group of irradiated patients we indeed detected significant differences. In addition, we did not observe changes in survival among non-irradiated patients. These data revealed that individuals with low expression of ECM-receptor genes presented improved response to IR, showing significantly increased survival when compared to patients with high expression of the ECM-receptor signature. We also found 7 genes for which augmented expression is associated with patients’ prognosis, regardless they were treated or not with IR (**Supplementary Figure 7**).

**Figure 6:**
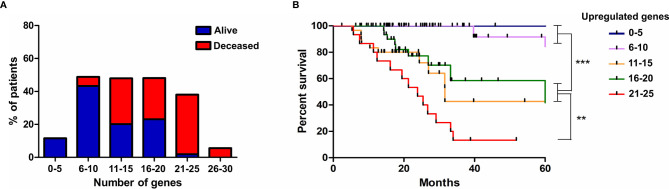
The progressive accumulation of up-regulated ECM-receptor interaction genes impacts the resistance of tumors to irradiation. **(A)** The number of genes with expression above the threshold defined by the ROC curve was evaluated in each patient submitted to IR treatment and six categories, with an increasing number of altered genes, were defined. The percentage of alive or deceased patients included in each category is indicated. **(B)** Kaplan Meier survival curves for LGG patients according to the categories described in **(A)**. Graphs were plotted with GraphPad Prism 4.0 software.

Finally, we evaluated if the progressive accumulation of up-regulated genes would impact the resistance of tumors to irradiation. For this, we defined six categories with an increasing number of altered genes, from up to 5 until 30, and evaluated the survival of patients, submitted to IR treatment, belonging to each class. We observed that among patients who survived for more than 5 years after diagnosis, 54% showed up to 10 overexpressed genes from the signature identified, 43% had up to 20, and only 2% exhibited more than 21 overexpressed genes (**Figure 6A**). However, among patients who died before 5 years of diagnosis, 5% showed up to 10 overexpressed genes, 52% revealed up to 20 up-regulated genes and 41% exhibited from 21 to 30 increased genes (**Figure 6A**). Kaplan-meier curves illustrate the survival of patients included in each category (**Figure 6B**). These data revealed that the number of genes from the ECM-receptor signature is a strong predictor of the response to radiotherapy, and suggests that patients displaying more than 20 overexpressed genes do not benefit from treatment.

## Discussion

Although radiotherapy is the main choice among the available treatments for GBM, it is known that this type of therapy induces high levels of genomic instability and considerable alterations in tumor microenvironment. An important consequence of RT is the remodeling of the extracellular matrix, promoting upregulation of several ECM proteins, such as: structural components, ligand-receptors, proteases and regulators of tumor cells adaptation. Altogether, these alterations culminate in increased survival, proliferation, migration, invasion and angiogenesis, supporting the prominent aggressiveness and the frequent recurrences of GBM (6). In this study, we evaluated irradiation-induced cell death levels and observed that the GBM cells with lower proliferation levels, U87MG and U138MG, are more resistant to ionizing radiation (IR) and present many overexpressed genes associated with the ECM-receptor interaction pathway. According to our observations, it has been shown that GBM cells are able to remodel the associated ECM by promoting coordinated alterations in cell adhesion, which were mediated mainly by molecules such as integrins and cadherins, and cell detachment, caused by ECM degrading proteases (28). On the other hand, cells with higher proliferation ratios, such as T98G and U251MG, were more sensitive to IR and presented upregulation of several genes involved in DNA damage response, whose expression was also positively correlated with MKI67. The phenotype observed in the radiosensitive cell lines might be explained by the elevated cellular proliferation, which leads to replicative stress and induces genomic instability, promoting constitutive activation of the DNA damage signaling (29). Thus, the additional stress exogenously promoted by irradiation could potentiate the intrinsic replicative stress of these cells, intensifying cell death ratios. Among the cell lines utilized in our work, U87MG and U251MG were widely used in former studies for intracranial xenograft implants and showed the capability of inducing highly invasive tumors, containing nuclear atypia, hypercellularity, pleomorphism, and angiogenesis (30). However, cell proliferation indexes from tumors generated by these two cell lines were not evaluated in a comparable manner. It was also demonstrated that transcriptional profiles are drastically modified when *in vivo* models are used (31). This phenomenon implicates that cell behavior and the associated expression patterns might be divergent from that observed in the 2D culture model explored here. However, the clinical relevance of the genes identified in our study as biomarkers of IR-responsiveness was corroborated by the correlation between their expression levels and the response of glioma patients to radiotherapy.

Within the ECM-receptor interaction pathway, integrins are noteworthy. Integrins have been shown to play important roles in tumor microenvironment, being involved in the control of cell survival, proliferation, migration and invasion, since they activate pathways like FAK (focal adhesion kinase) and PI3K/AKT (phosphoinositide 3-kinase/protein kinase B) (7). According to our data, deregulation in integrin signaling were formerly associated with cancer (32). Several integrin subunits are overexpressed during astrocytoma progression and showed competence to promote invasion, angiogenesis, and radioresistance (33). Among the molecular subtypes of GBM, mesenchymal tumors are considered the most invasive and angiogenic (34, 35). Coincidentally, this subtype demonstrated global overexpression of integrins compared to others (33), highlighting integrins as attractive therapeutic targets for mesenchymal GBMs.

Curiously, distinct integrins were altered in either sensitive or resistant cell lines, such as ITGA6, ITGA8, ITGAV, ITGB4 and ITGB8 overexpressed in the sensitive cells, and ITGA3, ITGA5, ITGA10, ITGA11, ITGB1 and ITGB7 increased in the resistant cell lines. Considering the set of integrins upregulated in radioresistant cell lines, ITGB1, ITGA5 and ITGA3 were previously reported as associated with IR resistance. ITGB1 stabilizes RAD51 thus, favoring DNA double strand breaks repair by homologous recombination. This is enabled by the reduction of proteasome-mediated RAD51 degradation *via* RING-1 (24). Additionally, ITGB1 physically interacts with EGFR and increases *in vitro* resistance of GBM cells to IR in an AKT phosphorylation dependent manner, and has been related to worse prognosis of GBM patients (25). When dimerized with ITGA5, ITGB1 also promotes greater resistance of GBM cells by regulating the levels of the anti-apoptotic proteins Survivin and PEA-15 (26). Here we also demonstrated that ITGB1 and ITGA5 protein levels were gradually upregulated when we treated GBM cell lines with ionizing radiation, indicating that these proteins are IR-responsive. Accordingly, it has been shown that GBM cells present ITGB1 upregulation after irradiation, as well as radiosensitization when ITGB1 was silenced (27). In addition, radiation-induced upregulation of ITGB1 has been demonstrated *in vitro* and *in vivo* in prostate cancer cells (36), as well as upregulation of ITGA5 on colorectal tumor cells after X-ray irradiation (37). Furthermore, the mRNA expression of the genes comprising the CD151-ITGA3-ITGB1 complex is correlated to lower survival rates in GBM patients probably as a consequence of the synergistic effect with the EGF/EGFR pathway, which gives GBM cells greater motility and invasion (38). Since several ECM components activate the PI3K/AKT pathway, we also decided to investigate whether radioresistance is a consequence of this activation. However, after inhibiting AKT and simultaneously irradiating the resistant cell lines, we did not observe considerable sensitization, thus indicating that the ECM-receptor genes found as overexpressed do not depend solely on AKT signaling to promote radioresistance in the cell lines here studied and that other pathways might be involved. Regarding the integrins upregulated in sensitive cells, we observed higher amounts of ITGA6, which is able to regulate CHK1 and cdc25 levels in primary cultures of GBM neurospheres and is therefore important for the ATR signaling (39). In addition, ITGA6 was reported to induce the expression of the ZEB1 transcription factor and, consequently, the FGFR1 proliferation inducer (target of the ZEB1/YAP1 complex) (39). Thus, it could be suggested that ITGA6 might have a role in reducing the replicative stress of highly proliferative cells.

Additionally, we have also found that 31 genes of the ECM-receptor interaction pathway, out of 83, correlated with poor responsiveness of patients to radiation treatment. Supporting our findings, some of the genes contained in this signature were previously described as correlated to radioresistance. COL1A1, ITGB4 and VTN have been associated with radioresistance in different types of cancer, such as nasopharyngeal carcinoma, esophageal squamous cell carcinoma and head and neck cancer (40–42). Furthermore, although not correlating to response to irradiation, recent studies have shown that COL1A1, ITGA7, ITGB3, ITGB4, HMMR and IBSP upregulation confer low survival rate to glioma patients (43–48). Importantly, we have shown that the majority of patients with up to 10 simultaneously upregulated genes survive more than 5 years, while for most of the patients with more than 10 upregulated genes the prognosis is remarkably worse, with an overall survival lower than 5 years. It is also relevant to emphasize that radioresistant cells usually presented receptors and the respective ligands simultaneously upregulated, differently from the sensitive cell lines that eventually showed upregulation of either the receptor or the corresponding ligand. Thus, our results indicated that the upregulation of at least 21 ECM-receptor interaction genes is important for the acquisition of a radioresistant phenotype, mostly when both receptor and ligand are included.

In conclusion, our results corroborate with recent studies that have shown the ECM-receptor interaction pathway as an important driver of glioma radioresistance. More importantly, we identified for the first time, new markers of the ECM pathway correlated with GBM IR-resistance, namely: ITGA2B, ITGA7, ITGB4, ITGB7, COL1A1, COL6A1, COL6A2, COL6A6, COL9A1, CD36, CHAD, COMP, GP1BA, GP5, GP6, HMMR, IBSP, LAMA1, LAMA3, LAMA4, LAMC2, LAMC3, SV2C, TNC, TNN, TNXB, VTN and VWF. Notably, the genes identified as correlated with IR responsiveness in individual cell lines were validated in clinical data as biomarkers of patient response to radiotherapy. Additionally, the cell lines studied here proved to be useful models for functional experiments involving mechanistic investigations regarding IR resistance and proliferation capacity, once they can be explored as representative of different adaptive states of tumor cells. This group of genes could also be explored for screening patients who would benefit from radiotherapy and, moreover, represent promising targets for the development of adjuvant therapies that will possibly improve the outcome of patients with highly radioresistant gliomas.

## Data Availability Statement

The original contributions presented in the study are included in the article **Supplementary Material**. Further inquiries can be directed to the corresponding author.

## Author Contributions

Investigation, RS, PS, CC, LM, RN, RA, GN, CS, JS, and FS. Conceptualization, RS and VV. Formal Analysis, RS. Writing – original draft, RS, RA, GN, LP, and VV. Writing – Review and Editing, VV. Supervision and Resources, RP, CM, WS, and VV. Funding Acquisition, WS and VV. All authors contributed to the article and approved the submitted version.

## Funding

This work was supported by Fundação de Amparo à Pesquisa do Estado de São Paulo (FAPESP) (grants #2013/13465-1 and #2018/05018-9 to VV and grant #2013/08135-2 to WS), and by the Programa de Apoio ao Desenvolvimento Científico from the Faculty of Pharmaceutical Sciences of Araraquara (PADC). This study was also financed in part by the Coordenação de Aperfeiçoamento de Pessoal de Nível Superior – Brasil (CAPES) - Finance Code 001. RS received a post-doctoral fellowship from CAPES (#88887.369191/2019-00). CAPES also supported LM, GN, and CS; CNPq (Conselho Nacional de Desenvolvimento Científico e Tecnológico) supported CC and RN; and FAPESP supported PS (#2016/12744-2 and #2018/22799-4) and RA (#2017/15208-7 and #2019/24335-8).

## Conflict of Interest

The authors declare that the research was conducted in the absence of any commercial or financial relationships that could be construed as a potential conflict of interest.

## Supplementary Material

Supplementary Figure 1 The enriched KEGG pathways in treatment-sensitive cells in comparison to resistant cells. All genes with padj ≤ 0,0001 and log fold change > 2 in T98G and/or U251MG cells compared to U87MG and/or U138MG cells were subjected to pathway analysis by KEGG. The pathways related to cell-extracellular matrix interaction were highlighted in red. A False Discovery Rate (FDR) ≤ 0.05 was used as a threshold to select significant pathways. Graphs were plotted with GraphPad Prism 4.0 software.

Supplementary Figure 2 Interaction map of de-regulated genes from the ECM-receptor pathway according to KEGG analysis. Genes shown in red are upregulated in each indicated cell line, whereas genes shown in black did not present expression alteration. The figure was adapted from KEGG analysis result.

Supplementary Figure 3 qPCR validation of the RNAseq data. The expression of the ITGA3, ITGA5, ITGA8, LAMB1 and LAMA2 genes was evaluated in U87MG, U138MG, T98G and U251MG cells by qPCR. The relative expression of target mRNAs was normalized by HPRT. The non-tumor astrocytes ACBRI371 cells were used as reference for relative expression calculations. Graphs were plotted with GraphPad Prism 4.0 software. *p < 0.05, **p < 0.001, and ***p < 0.0001.

Supplementary Figure 4 AKT inhibition sensitizes U251MG cells to ionizing radiation. **(A)** The RNAseq data was used to obtain the number of reads per AKT1, AKT2 and AKT3 transcripts in U87MG, U138MG, T98G and U251MG cells. **(B)** AKT expression level and its phosphorylation ratio in U87MG, U138MG, T98G and U251MG cells. **(C)** Analysis of cell death after AKT inhibition and treatment with ionizing radiation. U87MG, U138MG, T98G and U251MG cells were treated with GSK690693 for 4 hours and irradiated with 10 Gy. Graphs were plotted with GraphPad Prism 4.0 software. **p < 0.001, and ***p < 0.0001.

Supplementary Figure 5 Nucleotide Excision Repair, Base Excision Repair and Non-homologous End-joining pathways in GBM cell lines. Heatmap of Nucleotide Excision Repair, Base Excision Repair and Non-homologous End-joining genes in U87MG, U138MG, T98G, U251MG and U343MG cells.

Supplementary Figure 6 Patients selected for treatment with ionizing radiation have worse survival prognosis. Kaplan Meier survival curves for LGG patients treated or not treated with ionizing radiation. **p < 0.001. The P-value was obtained from a log-rank test. Graphs were plotted with GraphPad Prism 4.0 software.

Supplementary Figure 7 High levels of 7 ECM-receptor interaction transcripts are correlated with poor survival prognosis in radiation-treated and non-treated patients. Kaplan Meier survival curves for LGG patients according to the ECM-receptor interaction genes expression levels in the tumors. Patients were divided into four groups: i) untreated patients whose tumors expressed low levels of the analyzed gene; ii) untreated patients whose tumors expressed high levels of the analyzed gene; iii) irradiated patients whose tumors expressed low levels of the analyzed gene; iv) irradiated patients whose tumors expressed high levels of the analyzed gene. *P < 0.05, **p < 0.001, and ***p < 0.0001. The P-values were obtained from a log-rank test. Graphs were plotted with GraphPad Prism 4.0 software.

Supplementary Table 1 Genes identified as differentially expressed in the comparisons between IR-resistant and sensitive cells. All genes with padj ≤ 0.0001 and log fold change > 2 were selected as differentially expressed in each indicated comparison.

Supplementary Table 2 Enriched KEGG pathways in each comparison of IR-resistant *versus* sensitive cells. All genes with padj ≤ 0.0001 and log fold change > 2 or < -2 of each comparison were subjected to pathway analysis by KEGG.

Supplementary Table 3 Clinical and expression data of LGG patients used in survival analysis. The clinical data of patients from the lower-grade glioma cohort and expression levels of the selected ECM-receptor interaction genes were downloaded from The Cancer Genome Atlas (TCGA) database (https://www.synapse.org/#!Synapse:syn2812961) and used for survival analysis.

Supplementary Table 4 Number of receptors, ligands and pair of ligand-receptors upregulated in each GBM cell line. The receptors and ligands were considered upregulated when padj ≤ 0.0001 and log fold change > 2.

Supplementary Table 5 Correlation between de-regulated genes from different categories and MKI67 expression. Data were obtained from the cbioportal.org website. Spearman’s Correlation > 0.5 was used as a threshold.

Supplementary Table 6 Values of expression threshold determined as adequate to define high and low expression of each gene utilized in the survival analysis. The receiver-operating characteristic (ROC) curves for death incidence by time were used to determine the expression cutoff.
