# Supplementary figures and images for: Expression Profiling of Glioblastoma Cell Lines Reveals Novel Extracellular Matrix-Receptor Genes Correlated With the Responsiveness of Glioma Patients to Ionizing Radiation
Source: Front Oncol. 2021 May 25;11:668090. doi: 10.3389/fonc.2021.668090 (PMC8240593; doi:10.3389/fonc.2021.668090)

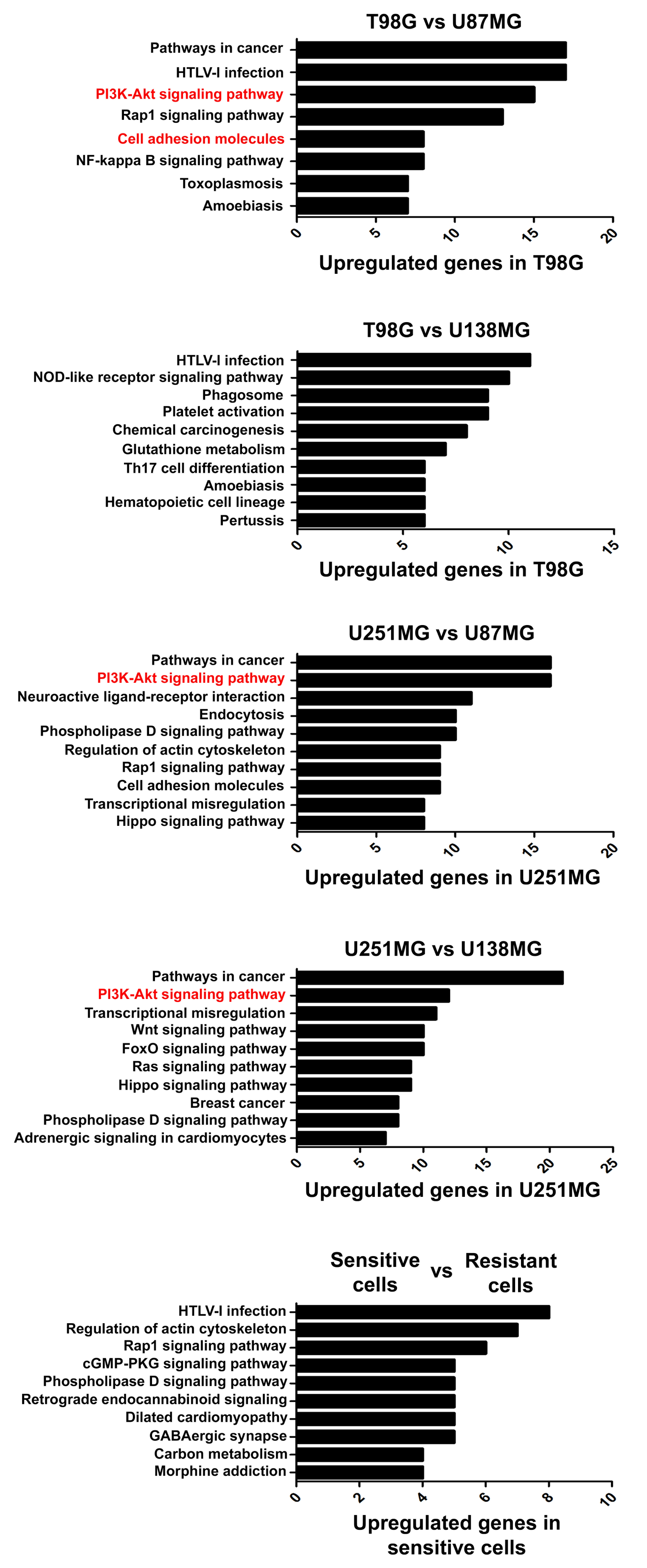

Supplement: Supplementary Figure 1 — The enriched KEGG pathways in treatment-sensitive cells in comparison to resistant cells. All genes with padj ≤ 0,0001 and log fold change > 2 in T98G and/or U251MG cells compared to U87MG and/or U138MG cells were subjected to pathway analysis by KEGG. The pathways related to cell-extracellular matrix interaction were highlighted in red. A False Discovery Rate (FDR) ≤ 0.05 was used as a threshold to select significant pathways. Graphs were plotted with GraphPad Prism 4.0 software. [file Image_1.tif]

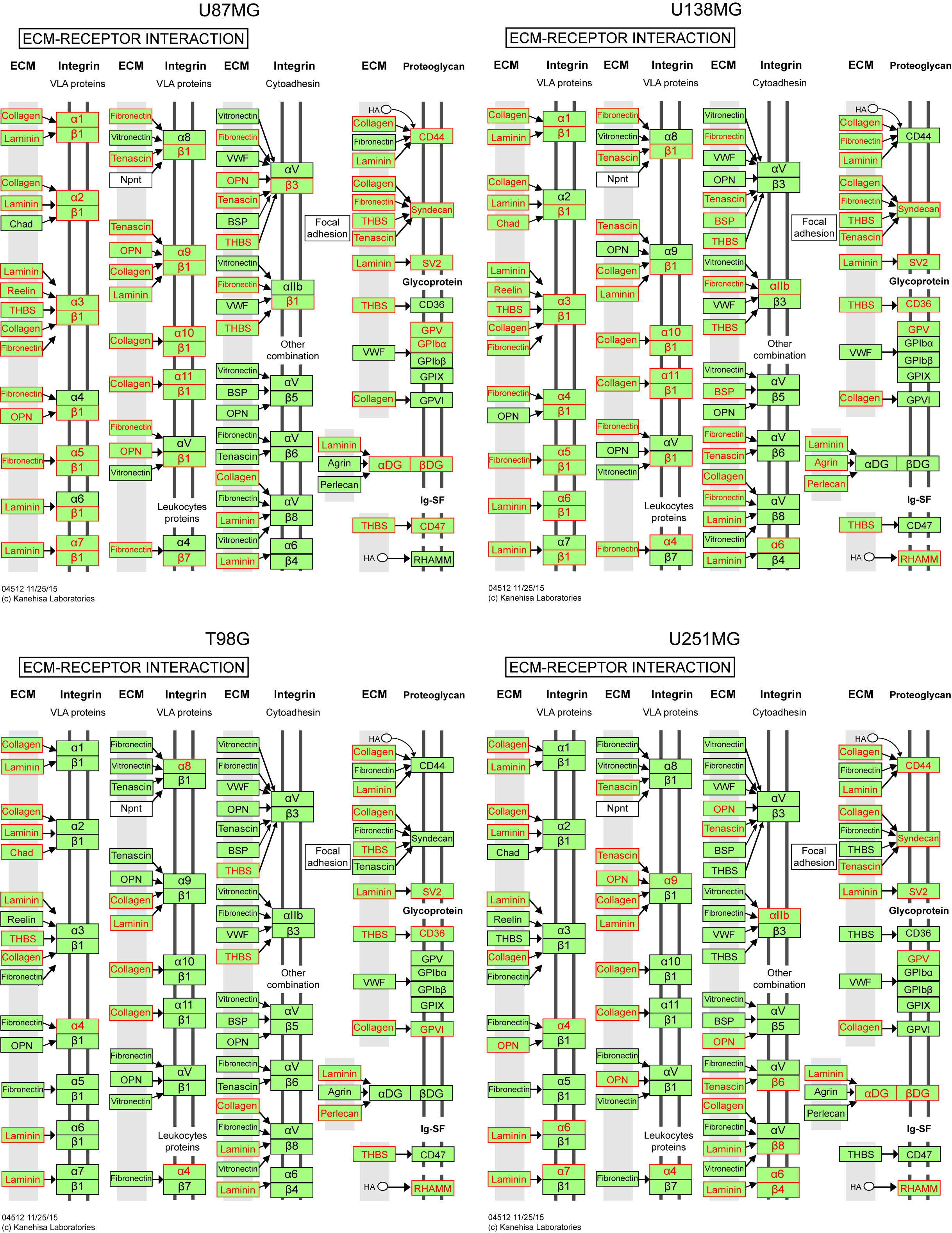

Supplement: Supplementary Figure 2 — Interaction map of de-regulated genes from the ECM-receptor pathway according to KEGG analysis. Genes shown in red are upregulated in each indicated cell line, whereas genes shown in black did not present expression alteration. The figure was adapted from KEGG analysis result. [file Image_2.tiff]

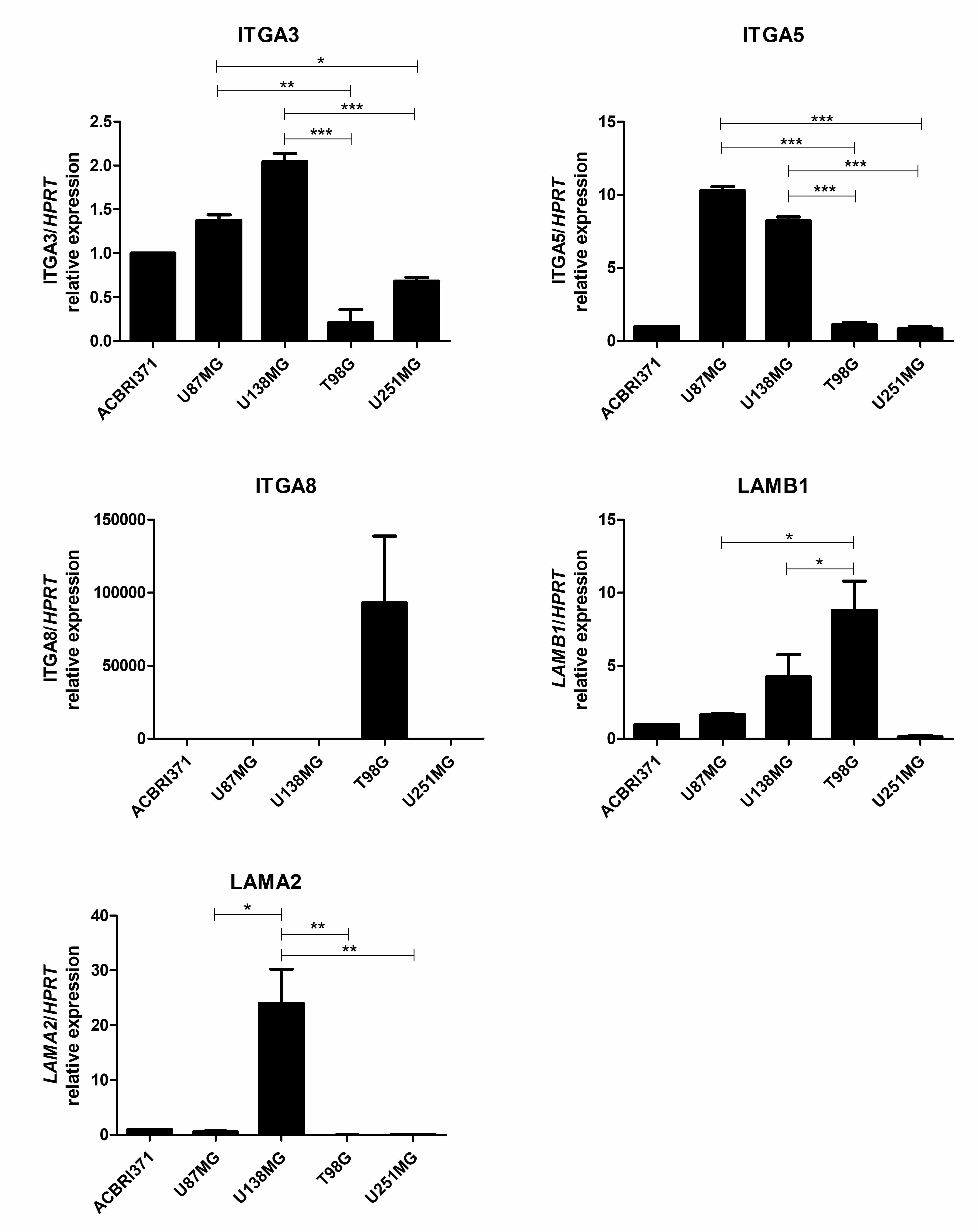

Supplement: Supplementary Figure 3 — qPCR validation of the RNAseq data. The expression of the ITGA3, ITGA5, ITGA8, LAMB1 and LAMA2 genes was evaluated in U87MG, U138MG, T98G and U251MG cells by qPCR. The relative expression of target mRNAs was normalized by HPRT. The non-tumor astrocytes ACBRI371 cells were used as reference for relative expression calculations. Graphs were plotted with GraphPad Prism 4.0 software. *p < 0.05, **p < 0.001, and ***p < 0.0001. [file Image_3.jpeg]

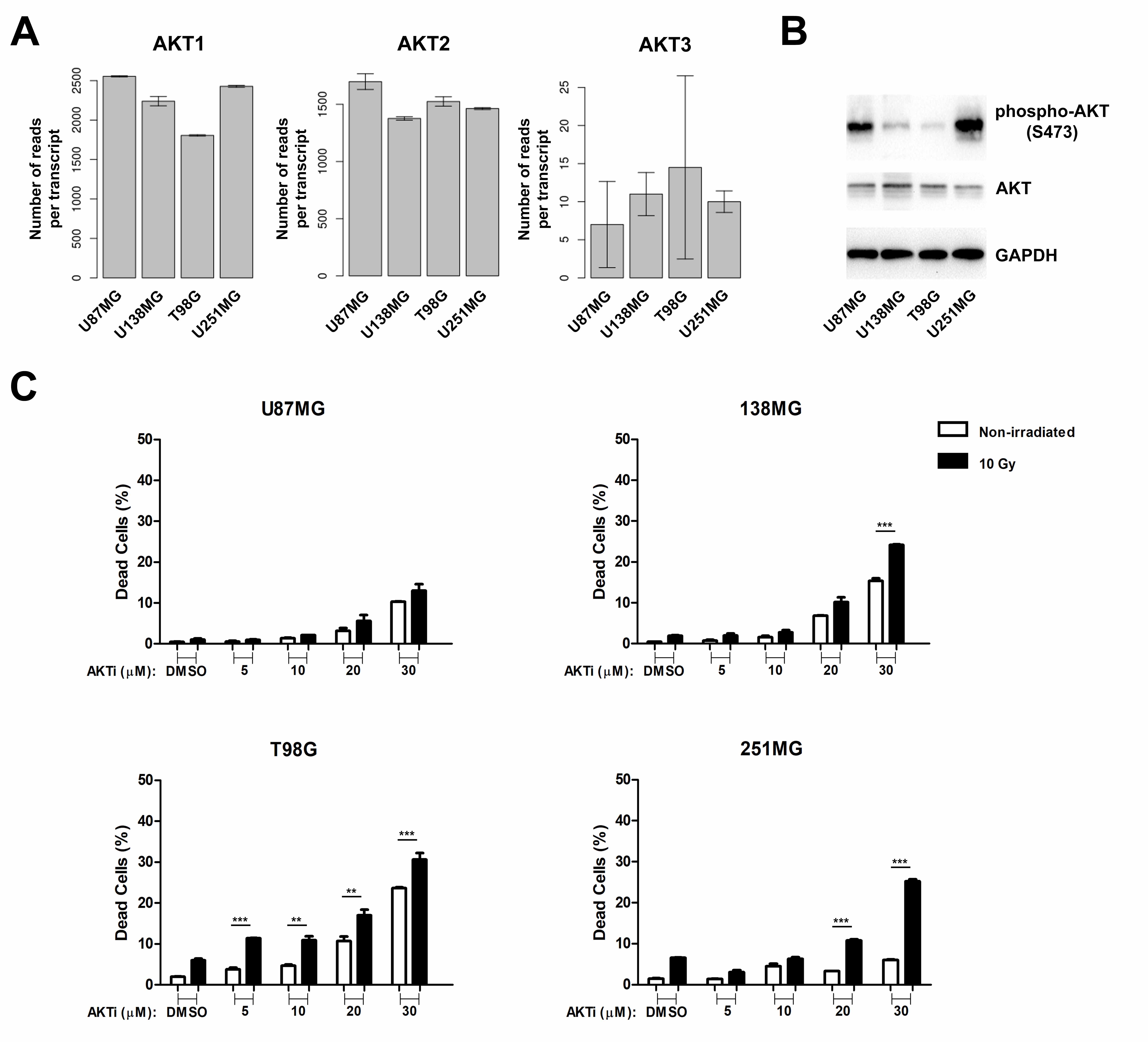

Supplement: Supplementary Figure 4 — AKT inhibition sensitizes U251MG cells to ionizing radiation. (A) The RNAseq data was used to obtain the number of reads per AKT1, AKT2 and AKT3 transcripts in U87MG, U138MG, T98G and U251MG cells. (B) AKT expression level and its phosphorylation ratio in U87MG, U138MG, T98G and U251MG cells. (C) Analysis of cell death after AKT inhibition and treatment with ionizing radiation. U87MG, U138MG, T98G and U251MG cells were treated with GSK690693 for 4 hours and irradiated with 10 Gy. Graphs were plotted with GraphPad Prism 4.0 software. **p < 0.001, and ***p < 0.0001. [file Image_4.jpeg]

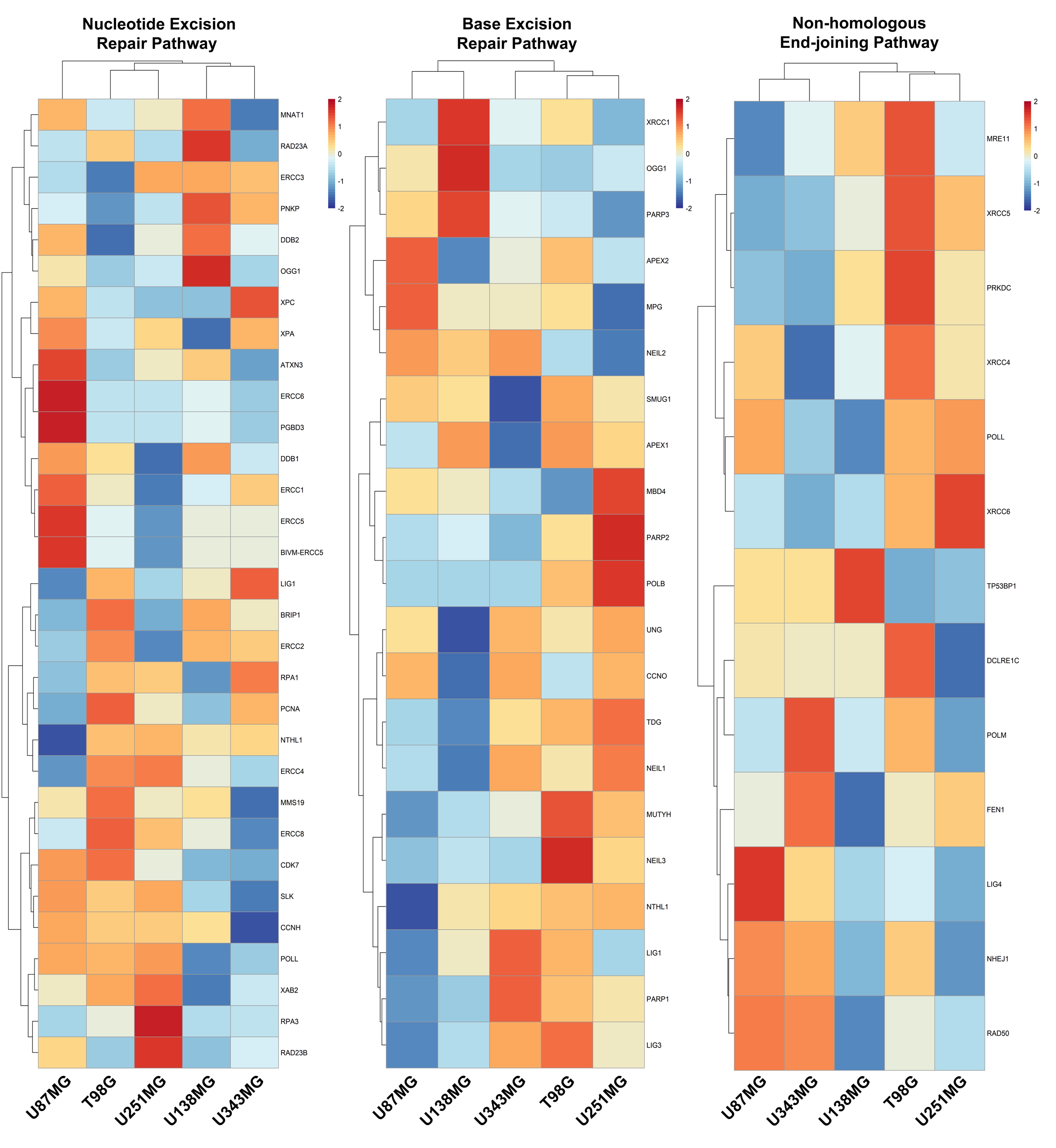

Supplement: Supplementary Figure 5 — Nucleotide Excision Repair, Base Excision Repair and Non-homologous End-joining pathways in GBM cell lines. Heatmap of Nucleotide Excision Repair, Base Excision Repair and Non-homologous End-joining genes in U87MG, U138MG, T98G, U251MG and U343MG cells. [file Image_5.tif]

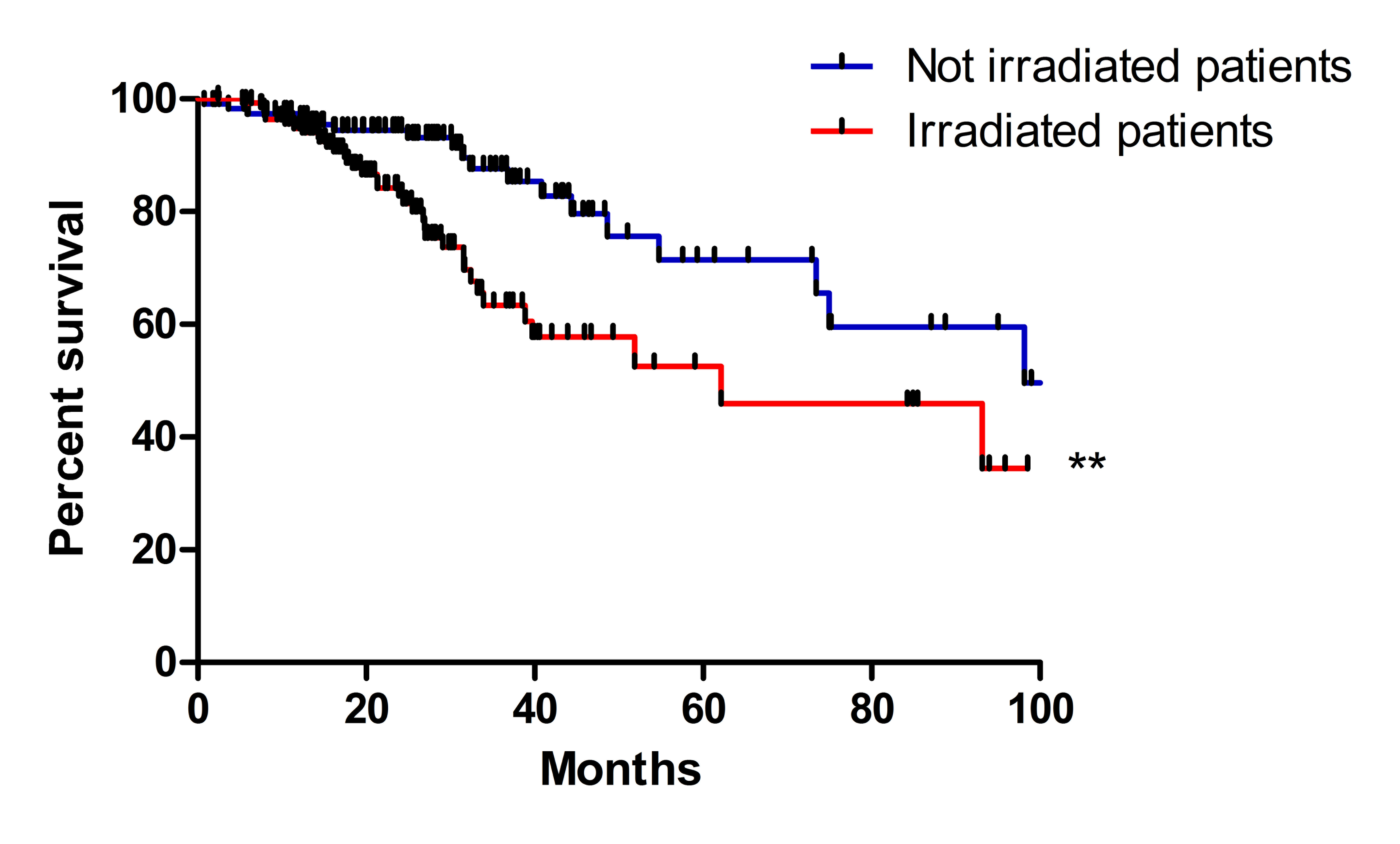

Supplement: Supplementary Figure 6 — Patients selected for treatment with ionizing radiation have worse survival prognosis. Kaplan Meier survival curves for LGG patients treated or not treated with ionizing radiation. **p < 0.001. The P-value was obtained from a log-rank test. Graphs were plotted with GraphPad Prism 4.0 software. [file Image_6.tif]

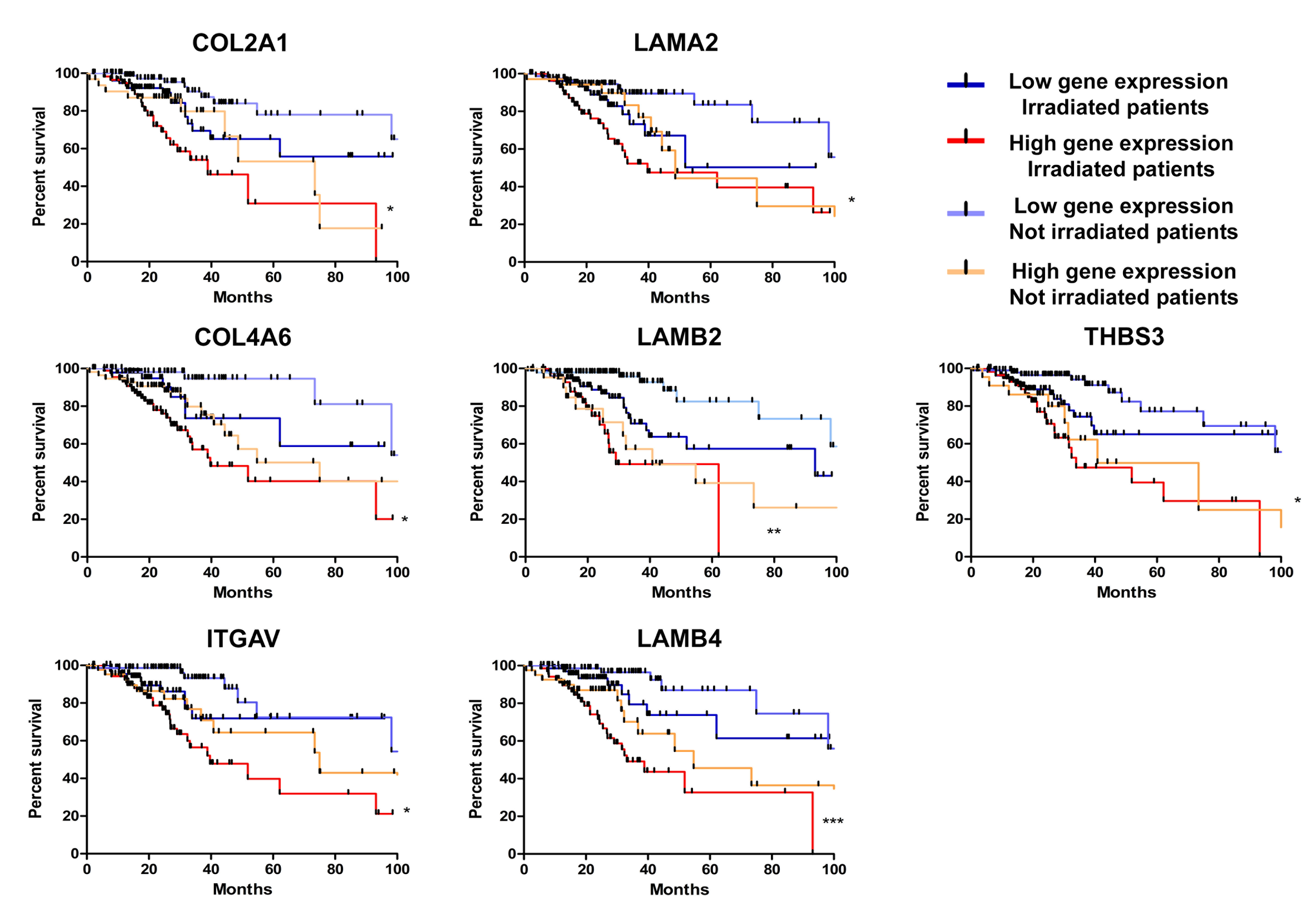

Supplement: Supplementary Figure 7 — High levels of 7 ECM-receptor interaction transcripts are correlated with poor survival prognosis in radiation-treated and non-treated patients. Kaplan Meier survival curves for LGG patients according to the ECM-receptor interaction genes expression levels in the tumors. Patients were divided into four groups: i) untreated patients whose tumors expressed low levels of the analyzed gene; ii) untreated patients whose tumors expressed high levels of the analyzed gene; iii) irradiated patients whose tumors expressed low levels of the analyzed gene; iv) irradiated patients whose tumors expressed high levels of the analyzed gene. *P < 0.05, **p < 0.001, and ***p < 0.0001. The P-values were obtained from a log-rank test. Graphs were plotted with GraphPad Prism 4.0 software. [file Image_7.tif]
